# Real‐time motion and retrospective coil sensitivity correction for CEST using volumetric navigators (vNavs) at 7T

**DOI:** 10.1002/mrm.28555

**Published:** 2020-11-09

**Authors:** Esau Poblador Rodriguez, Philipp Moser, Sami Auno, Korbinian Eckstein, Barbara Dymerska, Andre van der Kouwe, Stephan Gruber, Siegfried Trattnig, Wolfgang Bogner

**Affiliations:** ^1^ High Field MR Center, Department of Biomedical Imaging and Image‐guided Therapy Medical University Vienna Vienna Austria; ^2^ Neuroscience Center University of Helsinki Helsinki Finland; ^3^ Medical Physics and Bioengineering University College London London United Kingdom; ^4^ Athinoula A. Martinos Center for Biomedical Imaging Department of Radiology Massachusetts General Hospital Harvard Medical School Boston Massachusetts USA; ^5^ Christian Doppler Laboratory for Clinical Molecular MR Imaging Vienna Austria

**Keywords:** chemical exchange saturation transfer, coil sensitivity, inhomogeneities, motion correction, static magnetic field, ultra‐high field magnetic resonance

## Abstract

**Purpose:**

To explore the impact of temporal motion‐induced coil sensitivity changes on CEST‐MRI at 7T and its correction using interleaved volumetric EPI navigators, which are applied for real‐time motion correction.

**Methods:**

Five healthy volunteers were scanned via CEST. A 4‐fold correction pipeline allowed the mitigation of (1) motion, (2) motion‐induced coil sensitivity variations, $\Delta B_{1}^{‐}$, (3) motion‐induced static magnetic field inhomogeneities, ΔB_0_, and (4) spatially varying transmit RF field fluctuations, ${\mathrm{\Delta B}}_{1}^{+}$. Four CEST measurements were performed per session. For the first 2, motion correction was turned OFF and then ON in absence of voluntary motion, whereas in the other 2 controlled head rotations were performed. During post‐processing $\Delta B_{1}^{‐}$ was removed additionally for the motion‐corrected cases, resulting in a total of 6 scenarios to be compared. In all cases, retrospective ∆B_0_ and ‐${\mathrm{\Delta B}}_{1}^{+}$ corrections were performed to compute artifact‐free magnetization transfer ratio maps with asymmetric analysis (MTR_asym_).

**Results:**

Dynamic $\Delta B_{1}^{‐}$ correction successfully mitigated signal deviations caused by head motion. In 2 frontal lobe regions of volunteer 4, induced relative signal errors of 10.9% and 3.9% were reduced to 1.1% and 1.0% after correction. In the right frontal lobe, the motion‐corrected MTR_asym_ contrast deviated 0.92%, 1.21%, and 2.97% relative to the static case for Δω = 1, 2, 3 ± 0.25 ppm. The additional application of $\Delta B_{1}^{‐}$ correction reduced these deviations to 0.10%, 0.14%, and 0.42%. The fully corrected MTR_asym_ values were highly consistent between measurements with and without intended head rotations.

**Conclusion:**

Temporal $\Delta B_{1}^{‐}$ cause significant CEST quantification bias. The presented correction pipeline including the proposed retrospective $\Delta B_{1}^{‐}$ correction significantly reduced motion‐related artifacts on CEST‐MRI.

## 1 INTRODUCTION

Chemical exchange‐sensitive MRI techniques such as CEST^1^, ^2^, ^3^ and chemical exchange based on T_1ρ_ or T_2_ relaxation enhancement (ie, chemical exchange sensitive spin‐lock, CESL) provide an alternative chemically specific MRI contrast to MR spectroscopy, based on indirect detection of endogenous and exogenous molecules.^4^, ^5^, ^6^, ^7^, ^8^, ^9^, ^10^ In contrast to MRSI, the encoding of the spectral (chemically specific) dimension is slow. CEST and similar techniques rely on the comparison between images (ie, representing different spectral points) acquired at different time points. This makes these techniques particularly sensitive to motion, which is especially problematic for prolonged scans like in dynamic glucose enhanced (DGE) contrast experiments.^7^, ^10^, ^11^, ^12^, ^13^ The effect of motion‐induced artifacts in such dynamic CEST MRI sequences has been recently described by Zaiss et al^14^ at 3T. They reported motion‐induced artifacts in the same order of magnitude as the measured CEST effects (ie, 1% for every 0.6 mm of translation and every 7 Hz of shift in the static magnetic field; B_0_).

Several approaches have been proposed to mitigate motion artifacts in MR imaging and spectroscopy.^15^, ^16^, ^17^ They can be divided into prospective and retrospective methods. Prospective strategies track the displacement of the object and allow the update of the slice position and orientation in real‐time by adjusting magnetic field gradients and RF pulses. Head positions can be monitored in real‐time using navigators or by external MR‐compatible tracking systems (eg, optical). In contrast, retrospective correction methods like image registration or cross‐correlation have been developed and applied to high‐resolution structural and functional MRI. These require neither extra expensive hardware nor complicated sequence modification, but cannot account for all sources of artifacts.^18^

In functional MRI, retrospective motion correction using rigid body transformation has been applied to each volume in the functional series with good efficacy for small movements between acquisitions.^19^, ^20^ Motion correction based on registration has also been adopted in dynamic CEST and CESL MRI studies (eg, glucoCEST; glucoCESL).^6^, ^21^ In contrast, the use of prospective methods like prospective acquisition correction (PACE) showed a substantial improvement in mitigating the effects of larger head motion compared to such retrospective correction algorithms for functional MRI.^20^, ^22^ This facilitates its use in combination with real‐time motion correction methods based on navigators.^23^, ^24^, ^25^ In particular, the use of volumetric EPI navigators (vNavs) has reached a high level of accuracy in MRI (eg, up to 0.1 mm in translation and 0.2° in rotation) and has significantly improved the stability and reproducibility of MRSI measurements at 3T.^26^, ^27^, ^28^, ^29^, ^30^, ^31^ The application of real‐time B_0_/shim updates has been traditionally based on double‐echo navigators (de‐vNavs), although it can be replaced by 2 times faster mapping method based on single‐echo vNavs, as shown recently at 3T.^32^ Simegn et al applied these vNavs to correct motion and B_0_ alterations simultaneously in real‐time for the detection of glycogen at 3T (ie, glycoCEST). ^33^

However, they did not quantify remaining inhomogeneities in the main magnetic field ΔB_0_ after correction and did not attempt to apply any further post‐processing steps to compensate additional motion‐induced sources of artifacts. As presented recently by Moser et al,^34^ the use of real‐time B_0_ updates is still challenging at 7T.

At high (ie, 3T) and ultra‐high B_0_ (ie, 7T), the SNR and chemical specificity are improved, however, spatial B_0_ as well as transmit RF field ($B_{1}^{+}$) inhomogeneities across the FOV become more severe. ΔB_0_ causes frequency shifts and ${\mathrm{\Delta B}}_{1}^{+}$ amplitude deviations of the irradiated frequency‐selective CEST saturation pulses away from the targeted nominal values. This complicates quantification in CEST MRI.^35^, ^36^ A number of methods are available for their static correction,^37^, ^38^, ^39^, ^40^, ^41^ but these assume stable conditions throughout the acquisition of all Z‐spectral points of the CEST spectrum. This assumption is frequently violated, as Z‐spectral points are acquired at different time points. Only recently, studies proposing dynamic methods to mitigate temporal ∆B_0_ changes have emerged for CEST MRI. ^33^, ^42^, ^43^

Sources of motion‐induced artifacts are not limited to spatial displacements and B_0_ fluctuations, but also to temporal changes in $B_{1}^{+}$ and even more so in receiver coil sensitivities ($B_{1}^{‐}$). These $B_{1}^{‐}$ changes are a direct consequence of subject (eg, head) motion causing changes in the relative distance between investigated brain tissue and receive coil elements. An absolute signal change of *~*3% was reported for a 5° in‐plane head rotation within a 12‐channel head coil at 3T.^44^ This magnitude of signal deviation is comparable to the reduction of water signal because of saturation transfer and can, therefore, lead to significant bias in CEST contrast. To overcome this effect several strategies have been proposed, including adjustments of the receiver coil sensitivity maps in response to head motion for functional MRI or by a more robust definition of parameter estimation metrics for quantitative MRI (eg, in which the signal acquired by the coil is corrected by dividing it by a factor proportional to the coil receive sensitivity field).^44^, ^45^ In agreement with this, Boyd et al^5^ and Herz et al^8^ defined a chemical exchange‐sensitive DGE metric, which included a normalization parameter to account for the time‐varying coil sensitivities.

The aim of the present work was, therefore, to investigate whether the motion‐induced dynamic $B_{1}^{‐}$ changes can be accurately monitored using interleaved vNavs and whether this information can be used to compensate related artifacts on CEST‐weighted maps at 7T. Ultimately, this dynamic Δ$B_{1}^{‐}$ correction approach was combined with established real‐time rigid motion, dynamic ΔB_0_ and ${\mathrm{\Delta B}}_{1}^{+}$ correction techniques and its additional value was evaluated.

## 2 METHODS

### 2.1 Study design

The study was designed to compare a total of 6 scenarios. The first 3 static cases consider only negligible involuntary head movements during the measurements. They were performed to preclude possible undesired effects of the investigated motion/coil sensitivity corrections on the CEST contrast:

1. Static‐noMoco: no voluntary head motion and no vNav‐based dynamic correction
2. Static‐Moco: no voluntary head motion, but position and orientation of the CEST slice were updated in real‐time using vNavs^20^, ^26^, ^46^, ^47^
3. Static‐MoSensco: no voluntary head motion, but in addition to real‐time position/orientation updates also coil sensitivity ${\mathrm{\Delta B}}_{1}^{‐}$ correction was performed via vNav information.

The remaining 3 cases were evaluated to investigate the ability of our new correction pipeline to remove artifacts in CEST MRI that result from intended head motion:

- Motion‐noMoco: voluntary head motion, but no vNav‐based correction
- Motion‐Moco: voluntary head motion with vNav‐based real‐time motion correction^20^, ^26^, ^46^, ^47^
- Motion‐MoSensco: voluntary head motion with vNav‐based real‐time motion and retrospective coil sensitivity corrections.

**FIGURE 1:**
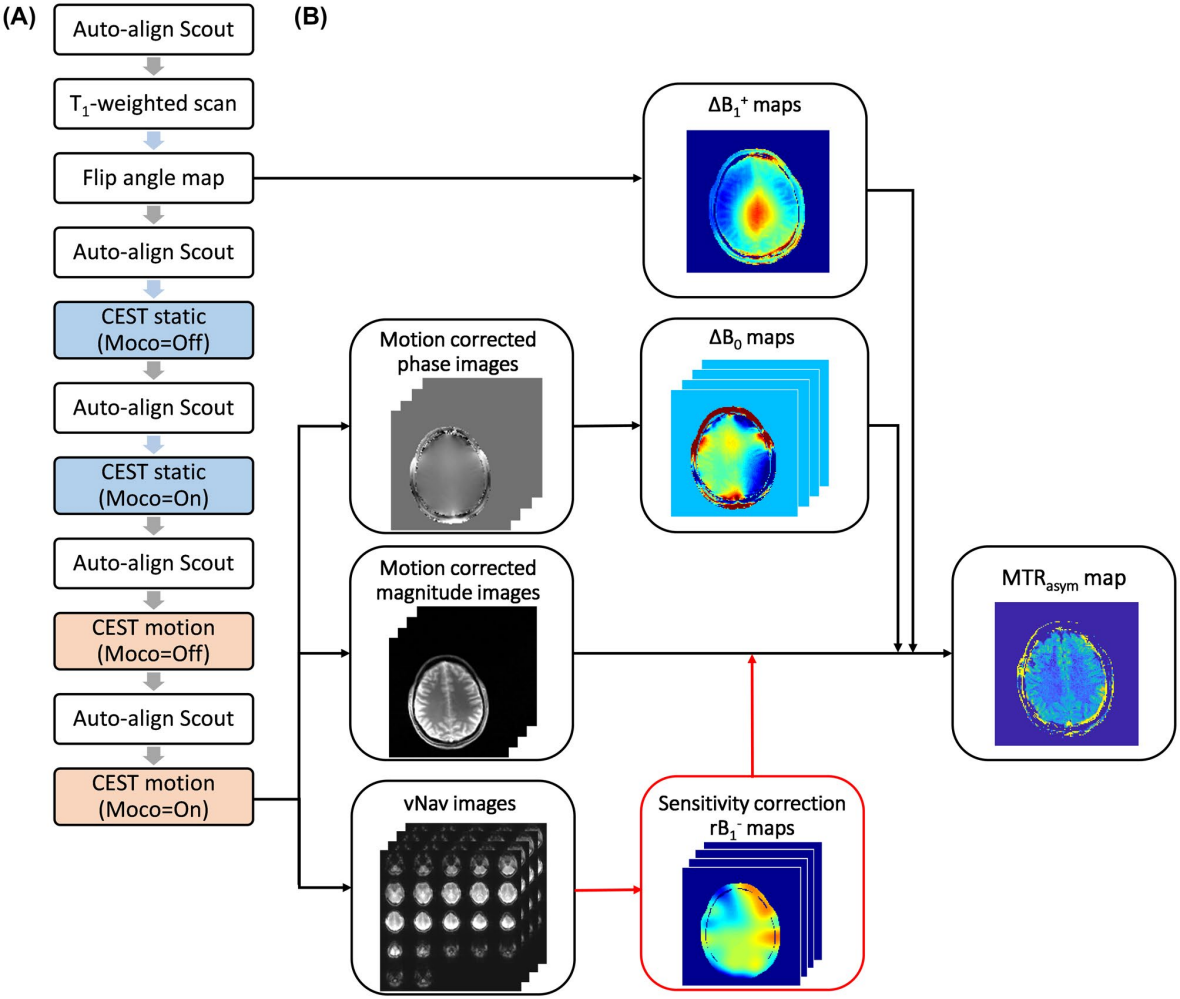
Chronological order of the experimental protocol followed to investigate the effect of motion and coil sensitivity corrections on CEST contrast (A). For the first 2 CEST scans (in blue) only involuntary motion was considered in contrast to the final 2 CEST acquisitions (in salmon color) for which intended head rotation was instructed. A scheme of the corrections implemented for the scenario Motion‐MoSensco (B) involves both: motion and coil sensitivity (${\mathrm{\Delta B}}_{1}^{‐}$; depicted in red) mapping. Static magnetic field (B_0_) and transmit field ($B_{1}^{+}$) inhomogeneities were additionally compensated to obtain artifact‐free MTR_asym_ maps

In all cases retrospective correction for ΔB_0_ (dynamically for each Z‐spectral point) and ${\mathrm{\Delta B}}_{1}^{+}$ were performed additionally as previously proposed.^41^, ^43^ The full measurement protocol is illustrated in Figure 1A. All sessions started with 2 pre‐scans: (1) a T_1_‐weighted 3D‐MP2RAGE sequence to position the center of the slice above the lateral ventricles with transversal orientation for subsequent scans, and (2) a pre‐saturated 2D single‐shot gradient echo (GRE) sequence to generate a flip‐angle map, needed for retrospective ${\mathrm{\Delta B}}_{1}^{+}$ correction. Subsequently, 4 CEST scans (with an embedded interleaved vNav sequence) were performed for the comparison of the 6 scenarios (ie, ${\mathrm{\Delta B}}_{1}^{‐}$ corrected scenarios had the same acquisition as the motion‐corrected ones). Prospective motion correction was turned OFF and ON respectively for scenarios without (Static‐noMoco; Static‐Moco + Static‐MoSensco) and with (Motion‐noMoco; Motion‐Moco + Motion‐MoSensco) intended head motion. To assure the same initial slice positioning for all CEST acquisitions, automatic alignment was performed referenced to a 3D MR brain atlas by running a vendor‐provided auto‐align scout at the beginning of the protocol and before each CEST scan. All volunteers were instructed to return to their initial head position after each CEST sequence.

Five healthy volunteers (4 males, 1 female; age 29.3 ± 4.4 years) were measured after approval by the local ethics committee and written informed consent was obtained. For each scan session, the protocol as described in Figure 1A was followed. During the last 2 CEST scans (corresponding to scenarios Motion‐noMoco and Motion‐Moco + Motion‐MoSensco), the subject was instructed to perform head rotations during the recovery time of measurements 8–13 of 34 (corresponding to Z‐spectral points at frequency offsets Δω = ±3.25 to ±2.75 ppm). The rotation started with the initial position looking upward followed by a head rotation toward the right‐hand side and finally remaining in this position until the end of the sequence. After each sequence, the initial head position was restored.

### 2.2 Acquisition

All examinations were performed on a whole‐body 7T MR system (Magnetom, Siemens Healthcare, Erlangen, Germany) with a birdcage transmit and a ^1^H 32‐channel receive head coil (Nova Medical, Wilmington, MA).

**FIGURE 2:**
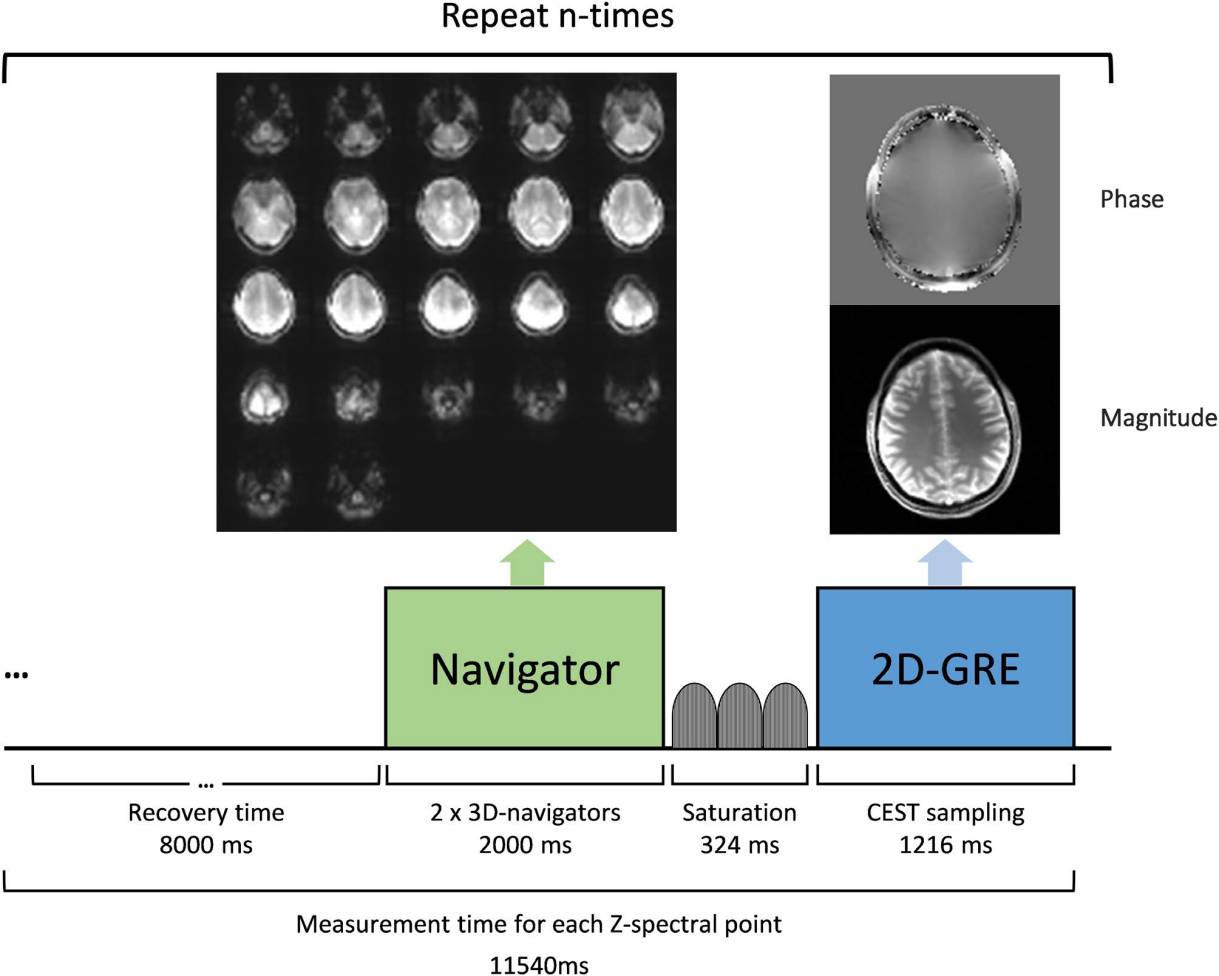
Overview of the sequence scheme for each CEST acquisition. During the CEST scan, the illustrated sequence was repeated *n* = 34 times: with 1 non‐saturated acquisition and 33 for each Z‐spectral point. Each acquisition is composed of 2 multi‐shot 3D EPI navigators (1 s each); 3 Gaussian RF saturation pulses (~0.32 s); 2D gradient‐echo readout (~1.22 s) and a delay to ensure T_1_ recovery of the water signal between different Z‐spectral points (8 s). The duration for acquisition is ~11.15 s. Note that the lengths of the illustrated blocks are not to scale

Each CEST scan included a total of 34 measurements (ie, 33 Z‐spectral points and 1 reference scan without suppression), each of them took ~11.5 s to acquire and consisted of (as shown in Figure 2): (1) a dual navigator block of 2000 ms, (2) a CEST saturation block of 324 ms, (3) an image sampling block of 1216 ms (ie, a train of gradient echoes), and (4) a recovery time of 8000 ms to allow full T_1_ relaxation between saturations blocks of ~11.2 s (ie, >5 × T_1_).^48^

1. The navigator block consisted of 2 consecutive vNavs. Their centers were ~1 s apart. The 1st vNav was mainly used to update the position of the 2nd vNav. This was important, because the 2nd vNavs were used to assess differences in coil sensitivity profiles, which requires that all of these vNavs are accurately co‐registered. In addition, larger displacements can be more accurately mitigated in 2 steps. This 2nd vNav was then followed by a ~324 ms saturation block. Therefore, both the prospective motion and retrospective coil sensitivity corrections of the CEST images were based on images generated by this 2nd vNav. All vNav images were acquired with the following parameters: 7 mm^3^ isotropic resolution; FOV of 224 mm^2^ with 22 slices; TE/TR = 6.1/11.0 ms; bandwidth = 4464 Hz/pixel; flip angle = 4°; echo train length = 16; water excitation only and slice partial Fourier 6/8.
2. The CEST saturation block included a train of 3 Gaussian RF pulses of amplitude 5.6 µT, 100 ms duration and 89% duty cycle with inter‐pulse RF crusher gradients. Z‐spectra were acquired with spectral resolution of 0.25 ppm over a frequency range of ±4 ppm. To reduce sensitivity to temporal changes, all Z‐spectral points were sampled in an alternating order from the periphery of the spectrum to its center (ie, −4, +4, −3.75, +3.75, −3.5, …, 0 ppm).
3. The image readout block was performed by a train of monopolar gradient‐echoes with centric k‐space reordering and dual‐echo readout to allow dynamic ΔB_0_ correction using the phase images of both TEs.^43^ The imaging parameters were: single slice; resolution of 1.8 × 1.8 × 5 mm^3^; FOV = 224 × 224 mm^2^; TE_1_/TE_2_/TR = 1.74/5.16/9.5 ms; readout bandwidth = 780 Hz/pixel and flip angle = 6°.

### 2.3 Data processing

All measured data (magnitude and phase images) were saved in DICOM format and processed retrospectively with an in‐house developed pipeline in MATLAB (R2017b, The MathWorks, Natick, MA). The processing pipeline is exemplified for the scenario of Motion‐MoSensco in Figure 1B.

For those scenarios for which real‐time motion correction was applied (ie, Static‐Moco; Static‐MoSensco; Motion‐Moco; Motion‐MoSensco), all CEST‐labeled images were additionally co‐registered with the first saturated image. In this way, the correction of residual artifacts possibly arising from involuntary subject movements taking place between the 2nd vNav and CEST image acquisitions was also considered.

CEST contrast was derived from the Z‐spectrum analysis, in which 33 Z‐spectral points were defined as:

(1) $$Z_{i}\left(\Delta \omega \right)=\frac{S_{i}\left(\Delta \omega \right)}{S_{0}\left(‐100\;\text{ppm}\right)},$$

in the frequency range −4 ≤ Δω ≤ +4 ppm. They were generated voxel wise from the GRE images by normalizing the pre‐saturated signal *S_i_*(Δω) with the reference *S*_0_(−100 ppm). For the scenarios Static‐MoSensco and Motion‐MoSensco, the Z‐spectra were modified to take the motion‐induced coil sensitivity into account, as:

(2) $$Z_{i}\left(\Delta \omega \right)=\frac{S_{i}\left(\Delta \omega \right)}{S_{0}\left(‐100\;\text{ppm}\right)\;\cdot \;S_{i}^{\mathrm{rel}}\left(\Delta \omega \right)}=\frac{S_{i}\left(\Delta \omega \right)}{S_{0}\left(‐100\;\text{ppm}\right)}\;\cdot \;rB_{1,i}^{‐}\left(\Delta \omega \right),$$

where the relative vNav signal *S_i_^rel^*(Δω) was used to estimate the CEST signal fluctuations at different frequency offsets. It was defined as the ratio:

(3) $$S_{i}^{\mathrm{rel}}\left(\Delta \omega \right)=\frac{S_{i}^{\mathrm{vNav}}}{S_{0}^{\mathrm{vNav}}},$$

where *S_i_^vNav^* is the magnitude signal corresponding to the *i*th measurement and *S_0_^vNav^* the initial vNav image as the reference. After *S_i_^rel^*(Δω) was computed for each voxel of the low resolution vNav volume, 3D smoothing and interpolation were performed to match the image resolution of CEST MRI and to derive *r* $B_{1}^{‐}$(Δω) for each measurement “*i*”. The smoothing step was performed by a robust discretized spline algorithm to provide reliable results even at brain’s boundaries (MATLAB function smoothn.m with smoothing parameter *S* = 0.5),^49^ as previously shown for distortion correction and coil combination.^50^, ^51^, ^52^, ^53^

Each Z‐spectral point was corrected for ΔB_0_ based on its own phase information using the dynamic method CEST‐GRE‐2TE proposed by Poblador Rodriguez et al^43^ and for ${\mathrm{\Delta B}}_{1}^{+}$ based on the 1‐point contrast Z‐B_1_ correction method proposed by Windschuh et al.^41^ Finally, the CEST contrast was calculated as the subtraction of Z‐values at symmetric Δω ranges of the spectrum:

(4) $$MTR_{\mathrm{asym}}\left(\Delta \omega \right)=Z_{i}\left(‐\Delta \omega \right)‐Z_{i}\left(+\Delta \omega \right),$$

### 2.4 CEST quantification: effect of motion and the proposed correction of motion‐induced artifacts

**FIGURE 3:**
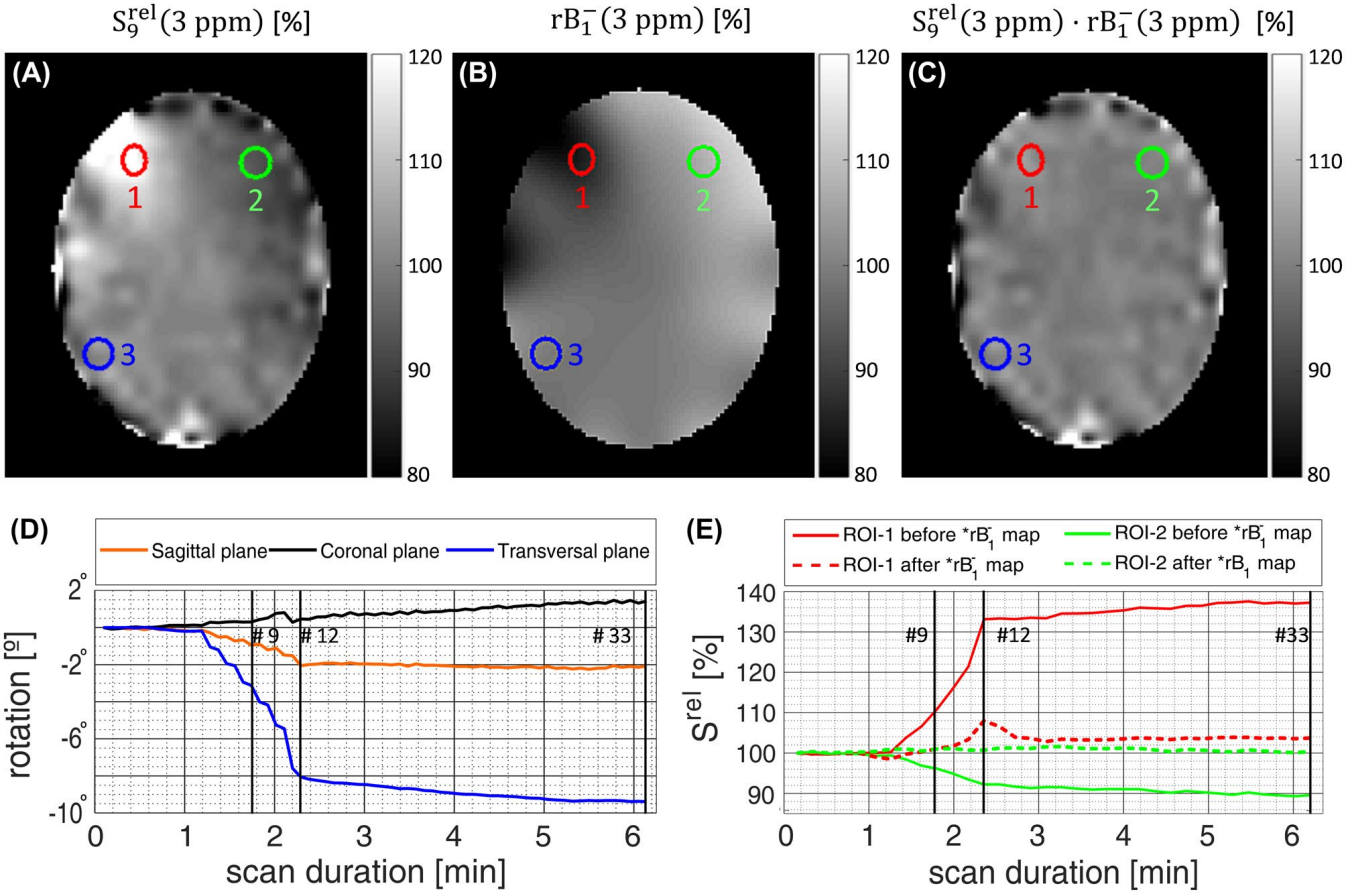
The up‐sampled vNav relative map (with respect to the initial vNav image prior to motion) corresponding to the 9th measurement S_9_^rel^(3 ppm) (A) is multiplied by the relative coil sensitivity correction map r$B_{1}^{‐}$(3 ppm) (B), resulting in corrected intensities close to ~100% with respect to the reference (C). The rotation along the transversal plane (in blue) for measurements 9th, 12th, and 33th reached 3.1° , 8.1°, and 9.4°, respectively for volunteer 4 (D). The evolution of the mean relative intensities S_9_^rel^ for ROIs 1 and 2 (red and green), located at the right and left frontal lobes respectively, show a mean error of 3.5% and −1.3% per degree of rotation for the 9th measurement (E). After multiplication with r$B_{1}^{‐}$ maps, these errors were reduced to 0.36% and 0.32% per degree of rotation. ROI‐3 location is displayed here for better understanding of future spatial analysis in which GM regions close to the rotation axes are compared to regions for which substantial displacements relative to the coil elements are expected (Figure 7)

For all vNav scans, 3D rigid motion (3 translation and 3 rotation) was estimated by PACE and sent back to the sequence. All image encoding gradients were updated once before each complete image acquisition (ie, 128 shots for the 2D‐GRE readout). The evolution of rotations over time is illustrated in Figures 3 and 5.

First, 2 elliptical regions of interest (ROIs) of 35.4 ± 4.8 and 37.0 ± 5.4 voxels were manually drawn in non‐saturated CEST images within gray matter (GM) in the right and the left frontal lobes of each volunteer. The impact of time‐varying coil sensitivities on the ROI‐averaged magnitude signal was assessed on up‐sampled vNav images (ROI‐1 and ROI‐2 in Figure 3) for all deliberate motion affected cases including those with (Motion‐Moco; Motion‐MoSensco) and without correction (Motion‐noMoco). r$B_{1}^{‐}$ correction maps were calculated as the inverse of smoothed and interpolated S^rel^ maps (Equation 2). The multiplication of the r$B_{1}^{‐}$ correction maps and the original vNav magnitude images was performed to validate the accuracy of this correction step with respect to motion (Figure 3E). The effect on the CEST‐labeled images was not considered at this point, to isolate the sensitivity changes from the concomitant effect of saturation.

**FIGURE 4:**
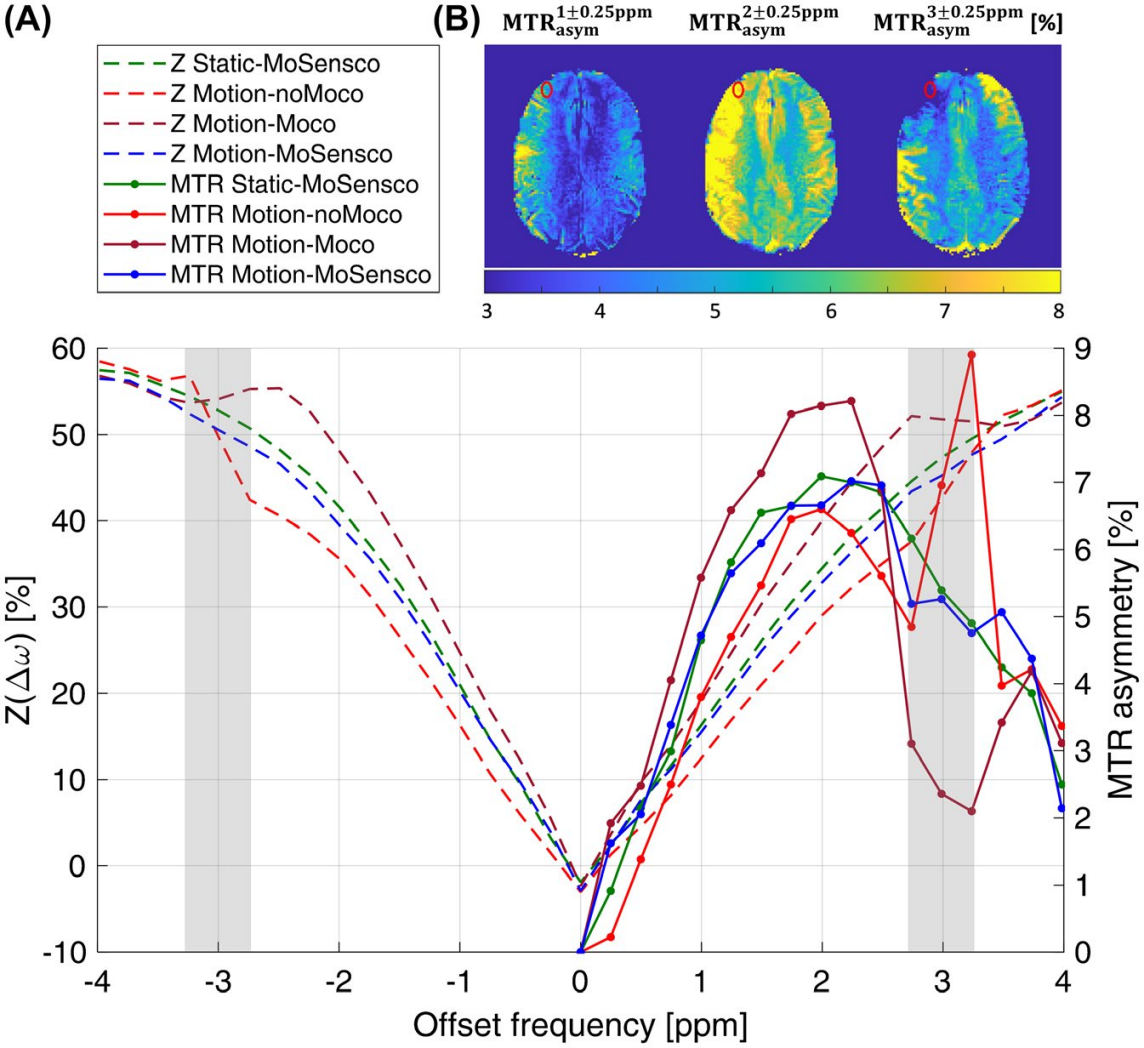
Effect of motion/corrections on CEST quantification: ROI‐averaged Z‐spectra (dashed) and MTR_asym_ (continuous) curves corresponding to volunteer 1 (A). The reference static scenario (Static‐MoSensco in green) is compared with: (1) the distortion caused by a deliberate total rotation of ~7° (Motion‐noMoco in red) performed during the acquisitions within the grey frames; (2) update of the position and orientation of the CEST slice in real time (Motion‐Moco in brown); and (3) additional coil sensitivity compensation (Motion‐MoSensco in blue). MTR_asym_ maps at 1,2,3 ± 0.25 ppm are shown for the scenario Motion‐Moco (B), where the analyzed ROI located in GM of the right frontal lobe is depicted in red

**FIGURE 5:**
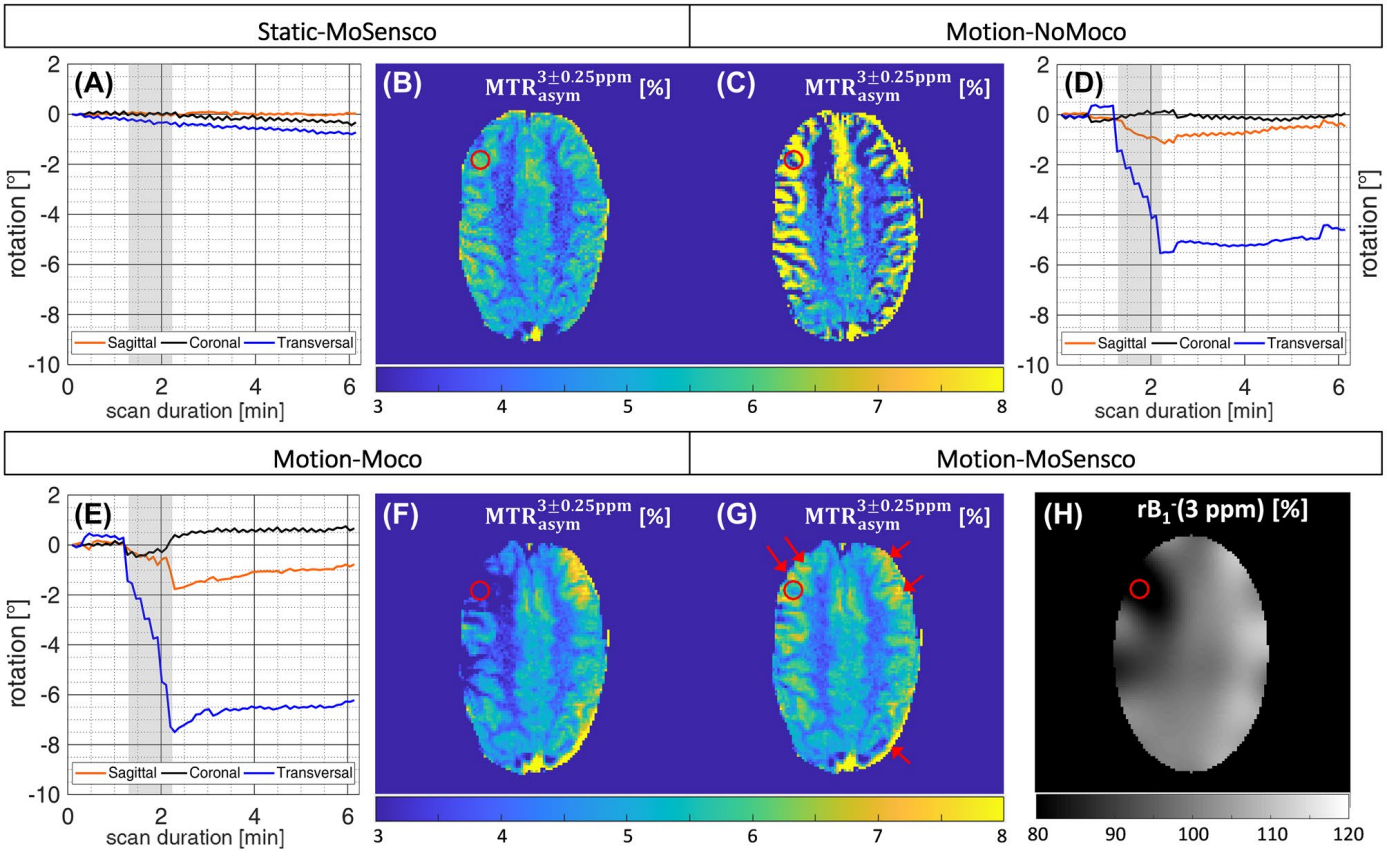
Comparison of spatial induced artifacts induced by head rotations on CEST contrast for volunteer 2. The MTR_asym_ (3 ± 0.25 ppm) weighted map corresponding to scenario Static‐MoSensco was considered as reference (B). An involuntary rotation in the transversal plane of 0.28 ± 0.05° (blue curve) was observed during the acquisition of the respective 6 Z‐spectral points (in the time range *t* = [1.4–2.3] min) (A). Strong hyper/hypo‐ intense contrast values around tissue interfaces were apparent in the MTR_asym_ map (C) for an intended transversal rotation of 3.17 ± 1.32° (D) when no motion was corrected (scenario Motion‐noMoco). After real‐time update of the CEST slice (and additional image co‐registration for scenario Motion‐Moco), coil sensitivity artifacts became predominant in the MTR_asym_ map (F). As expected, erroneous hypointense CEST contrast spots emerged in the right frontal lobe (left‐hand side of the map) and hyperintense ones in the left frontal lobe (right‐hand side of the map) for a rotation of 3.90 ± 2.06° in the transversal plane. The proposed dynamic ${\mathrm{\Delta B}}_{1}^{‐}$ correction method, implemented as multiplication of the CEST images with the relative coil sensitivity correction maps (H), resulted in a fully corrected CEST weighted map (G). Residual motion artifacts were still present close to the brain borders (pointed to by red arrows)

Second, the influence of motion on CEST quantification was evaluated on Z‐spectra and magnetization transfer ratio curves with asymmetric analysis (MTR_asym_) for scenarios with intended motion (ie, Motion‐noMoco; Motion‐Moco; Motion‐MoSensco) relative to the Static‐MoSensco case (ROI‐1 in Figure 4). Finally, the spatial effects of motion and subsequent corrections were assessed for the same 4 scenarios by evaluating the MTR_asym_ maps at Δω = 3 ± 0.25 ppm. This frequency range should be most affected as the rotations took place during the acquisition of these Z‐spectral points (Figure 5).

### 2.5 Statistical analysis

All statistical analyses were conducted using SPSS Statistics (24, IBM, Armonk, NY) for which ROIs at 3 locations were defined for each of the 5 participants. In addition to the 2 previously mentioned ROIs in frontal GM regions, a third one 36.2 ± 3.9 voxels in size was placed in GM located close to the rotation axes at the back of the head. These 3 ROIs were representative for (1) a region with decreasing distance to the coil elements (frontal right‐hand side of the coil; ROI‐1) while rotation takes place, (2) a region with increasing distance (frontal left‐hand side; ROI‐2), and (3) a region with mild positional variation relative to the receive coil elements (posterior; ROI‐3). All ROIs are depicted in Figure 3.

To exclude potential bias from the investigated motion and coil sensitivity corrections, the static scenarios (ie, Static‐noMoco; Static‐Moco; Static‐MoSensco) were first considered and all voxels belonging to the ROIs defined in these 3 locations were selected. A one‐way analysis of variance (ANOVA) was computed considering MTR_asym_ contrasts of each voxel of the selected ROIs as the dependent variable and the different scenarios as the independent variable. The MTR_a__sym_ maps were calculated for 3 frequency ranges Δω = 1, 2, 3 ± 0.25 ppm. *P* < 0.05 was considered statistically significant. Bonferroni post‐hoc tests were additionally executed for those comparisons in which significant differences were found.

To assess the benefit of the proposed fully corrected scenario under voluntary motion (Motion‐MoSensco), MTR_asym_ distributions among all scenarios were compared using histograms and Games‐Howell post‐hoc tests were calculated. In this way, the null hypothesis assumed no difference with respect to both: (1) static scenarios, meaning a successful restoration of the CEST‐weighted maps despite the voluntary rotations; and (2) non/partially corrected scenarios (ie, Motion‐noMoco and Motion‐Moco), implying a minor correction step toward artifact‐free CEST MRI.

**FIGURE 6:**
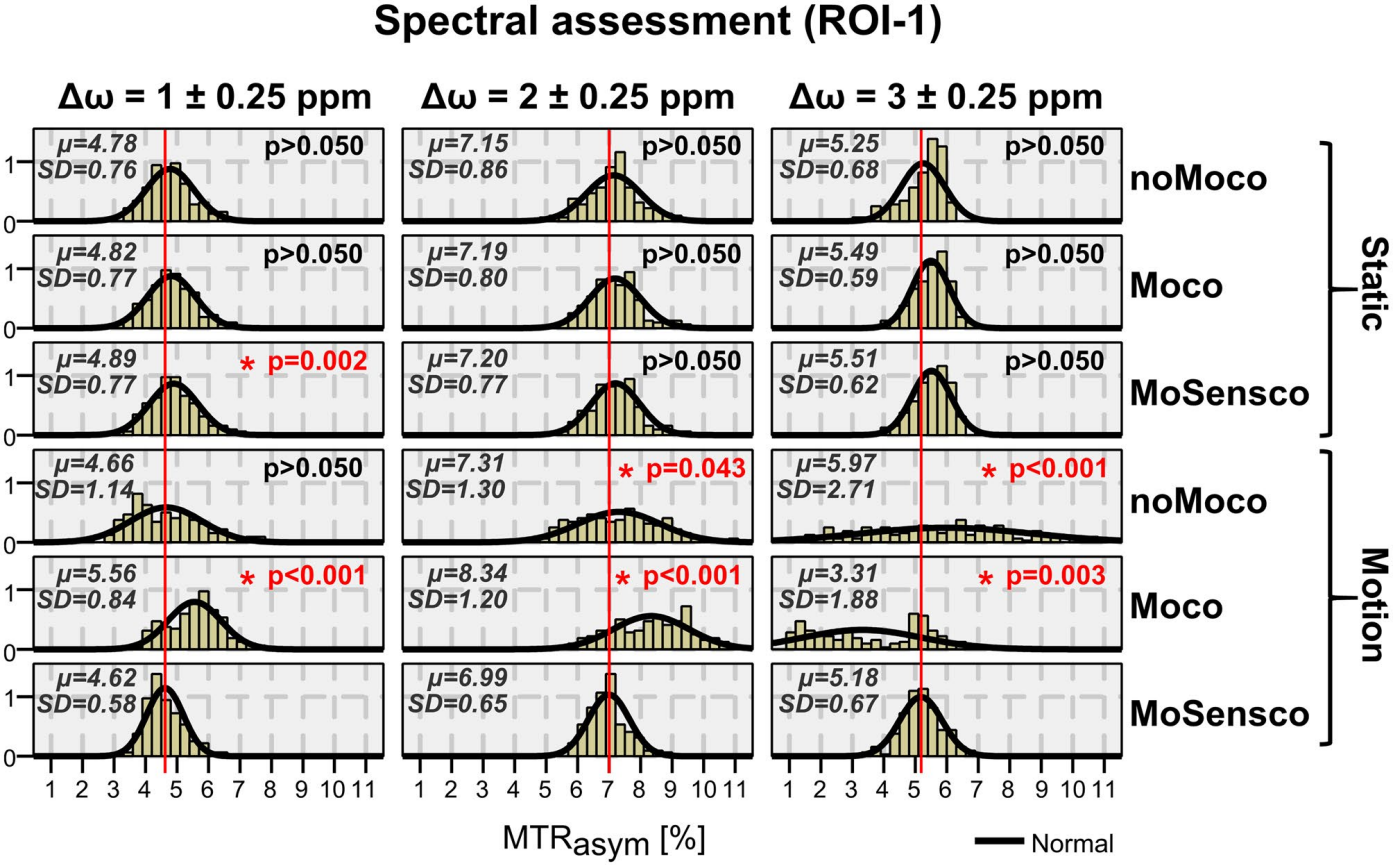
Histograms show the distribution of MTR_asym_ values within ROI‐1 (right frontal lobe) for different scenarios (rows; ie, static/motion with or without correction) and contrasts calculated at Δω = 1, 2, 3 ± 0.25 ppm (columns). Mean and SD of MTR_asym_ distributions are shown. *P*‐values resulting from Games‐Howell post‐hoc test for one‐way ANOVA with respect to the final scenario “MoSensco” are highlighted in red for scenarios with significant difference (ie, *P* < 0.05). This figure illustrates that in presence of voluntary motion, dynamic coil sensitivity correction proved to be a significant step against motion‐induced artifacts regardless of the frequency of interest by restoring the expected narrow distribution of MTR_asym_ value within ROI‐1

**FIGURE 7:**
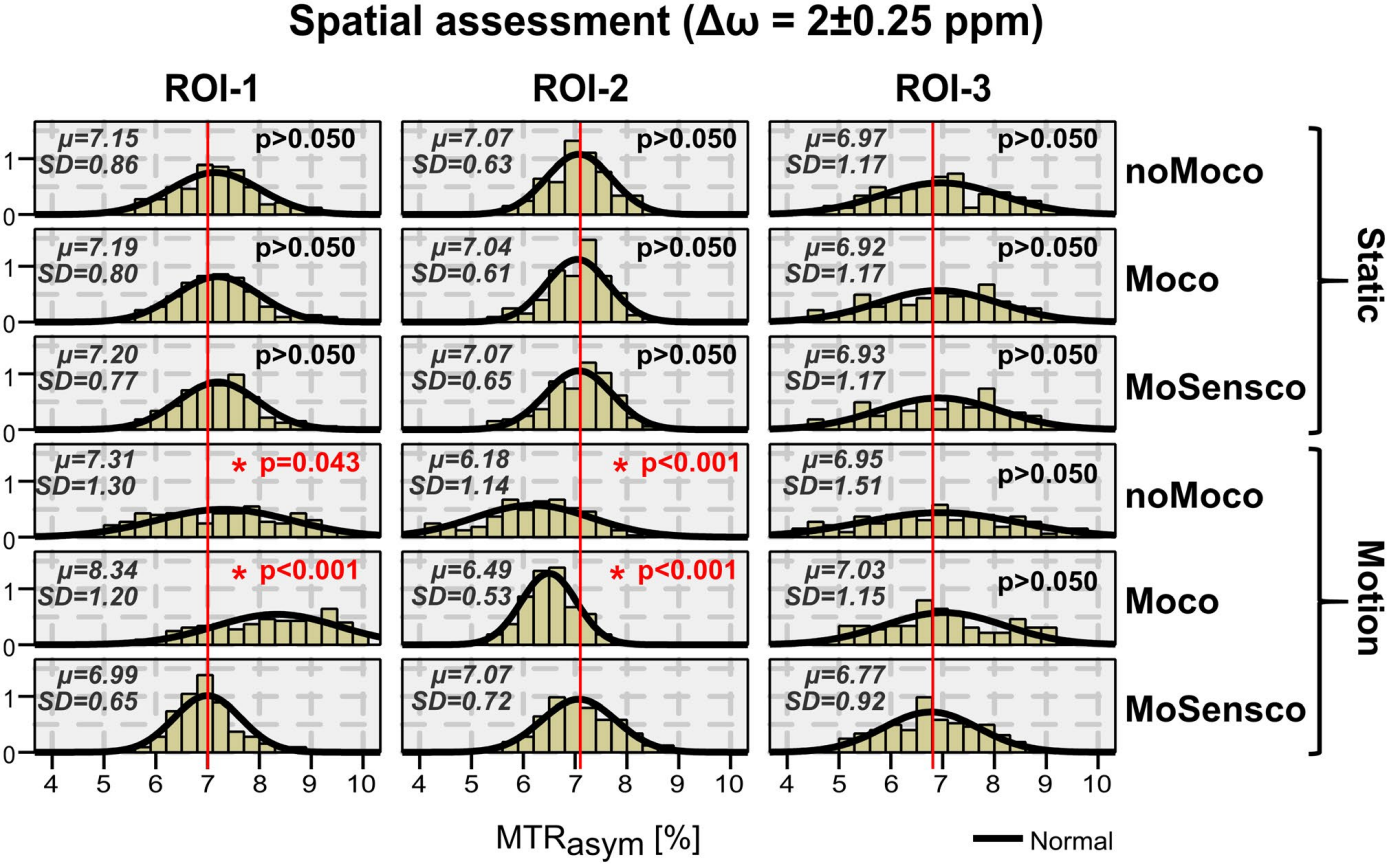
Histograms show the distribution of MTR_asym_ values within ROI‐1, 2, and 3 (columns) for different scenarios (rows; ie, static/motion with or without correction) when contrasts were calculated over a single frequency range Δω = 2 ± 0.25 ppm. The ROIs were defined as follows (from left to right, see also Figure 3): ROI‐1, GM regions far from the axes of rotation and moving closer to the coil elements as rotation takes place; ROI‐2, GM regions far from the axes of rotation and moving away from the coil elements as rotation takes place; and ROI‐3, GM regions close to the rotation axes, for which mild displacements relative to the coil elements are expected. Mean and SD of MTR_asym_ distributions are shown. *P*‐values resulting from Games‐Howell post‐hoc test for one‐way ANOVA with respect to the final scenario “MoSensco” are highlighted in red for scenarios with significant difference (ie, *P* < 0.05). As expected, regions experiencing a greater displacement with respect to the coil elements (ROI‐1 and ROI ‐2) improved significantly with the proposed dynamic coil sensitivity correction, whereas ROI‐3 did not

To investigate how motion‐induced artifacts bias the distribution of MTR_asym_ values within specific ROIs and how corrections influence this behavior across volunteers, a region located in the right frontal lobe (ROI‐1) was selected for each subject and histograms were presented in Figure 6 for each scenario and frequency range (ie, Δω = 1, 2, 3 ± 0.25 ppm). Finally, we investigated how the distribution of MTR_asym_ values differs for different brain regions (ie, ROIs) while fixing the frequency range to Δω = 2 ± 0.25 ppm. The respective histograms for each scenario and selected ROI are shown in Figure 7. ROI‐1 was representative for a region approaching the coil elements, ROI‐2 for a region moving away, and ROI‐3 for a region with slight positional changes.

## 3 RESULTS

### 3.1 CEST quantification: effect of motion and the proposed correction of motion‐induced artifacts

Figure 3 illustrates the impact of motion on vNav images and efficacy of the smoothing step needed for ${\mathrm{\Delta B}}_{1}^{‐}$ correction for 1 volunteer. The 6 individual rotations in the transversal plane were typically kept <1° except for the one preceding the 12th measurement in which ~2° was observed, resulting in an accumulated rotation of 3.1°, 8.1°, and 9.4° for the 9th, 12th (end of the voluntary rotation), and 33th (last) measurement, respectively. At these time points, the averaged relative signal S^rel^ within ROI‐1 in the right frontal lobe increased by +10.9%, +33.1%, and +37.3% relative to its starting value. These relative intensity variations were mitigated to +1.1%, +8.0%, and +3.7% (for measurements 9th, 12th, and 33th) after multiplication with r$B_{1}^{‐}$ correction maps. The signal change resulting from the described rotation was smaller for ROI‐2, defined in the left frontal lobe, where the relative signal decreased by −10.4% at most for the last measurement. After correction, the highest observed relative signal intensity change was +1.6%.

In case of voluntary head motion, but no correction (ie, Motion‐noMoco), CEST quantification was, therefore, very sensitive to head movements for Z‐spectral points acquired during the intended rotation (ie, Δω = 3 ± 0.25 ppm). As shown in Figure 4 for 1 subject (representative for all volunteers), the update of the position and orientation of the CEST slice (scenario Motion‐Moco) unexpectedly resulted in stronger MTR_asym_ curves deviations with respect to the reference scenario (Static‐MoSensco) not only at ~3 ppm but also at ~2 ppm.

The smaller deviation in Motion‐noMoco can be explained by the fact that the Z‐spectra and MTR_asym_ curves were averaged within an ROI. In this way, once the motion artifacts (characterized by strong hyper/hypo‐intensities around tissue boundaries, as seen in Figure 5C) are compensated, the artifacts originated by the varying coil sensitivities became predominant. They consisted of hypointense contrast values at ~3 ppm and hyperintensities at ~2 ppm for ROI‐1 located in the right frontal lobe. These artifacts were reduced by the coil sensitivity compensation (ie, Motion‐MoSensco), resulting in an MTR_asym_ curve that was much more similar to that of the static reference case.

The spatial appearance of motion‐induced artifacts and the correction efficacy of single correction steps are presented in Figure 5 for a representative volunteer. The motion displacement artifacts, produced by an intended rotation toward the right‐hand side in the transversal plane, presented strong over‐ or under‐estimation of the CEST contrast in the interfaces between GM/white matter tissues (ie, Motion‐NoMoco). After real‐time motion update of the CEST slice and subsequent image registration (ie, Motion‐Moco), the artifacts arising from temporal changes in receiver coil sensitivities (${\mathrm{\Delta B}}_{1}^{‐}$) became predominant. They presented hypointense CEST contrast for regions moving toward the coil elements (right lobe) and hyperintensities for regions moving away (left lobe) for Δω = 3 ± 0.25 ppm. Extra residual artifacts were still present close to the brain borders. The final dynamic ${\mathrm{\Delta B}}_{1}^{‐}$ correction step (ie, Motion‐MoSensco) successfully compensated the coil sensitivity‐induced distortions. The resulting fully corrected MTR_asym_ (3 ± 0.25 ppm) map presented contrast much more similar to the static case (ie, Static‐MoSensco) despite an observed rotation of 3.90 ± 2.06° in the transversal plane during the acquisition of the corresponding 6 CEST images (Δω = ±2.75 to ±3.25 ppm).

An additional quantitative evaluation of performance of the proposed processing pipeline can be found in the Supporting Information. Here, the global quality assessment metrics SSIM (structural similarity) and MSE (mean squared error) are derived and presented for each volunteer (Supporting Information Tables S1 and S2). In addition, SSIM quality maps have been included in Supporting Information Figure S1 for visual assessment of local structural differences among scenarios. This assessment serves as a check over entire MTR_asym_ maps and reveals that locally computed analysis (eg, ROI‐based) are more appropriate than global indexes (ie, over entire images) to evaluate the restoring capability of the proposed ${\mathrm{\Delta B}}_{1}^{‐}$ correction step.

### 3.2 Statistical analysis

No significant effects were found at Δω = 1,2 ± 0.25 ppm when comparing the MTR_asym_ values among the static cases (ie, Static‐noMoco, Static‐Moco and Static‐MoSensco). However, there were significant differences in the Δω = 3 ± 0.25 ppm spectral range (*F* [2,1628] = 13.76, *P* < 0.001). Post‐hoc multi‐comparisons at Δω = 3 ± 0.25 using Bonferroni correction indicated that the mean MTR_asym_ for the scenario Static‐noMoco (M = 5.25, SD = 0.65) was significantly different to that obtained for the Static‐Moco (M = 5.42, SD = 0.67) and Static‐MoSensco (M = 5.45, SD = 0.68) cases. This could be because of uncorrected involuntary movements in scenario Static‐noMoco.

To investigate the spectral effect of the motion‐induced artifacts and its corrections, histograms of MTR_asym_ values within ROI‐1 at frequency offsets Δω = 1, 2, 3 ± 0.25 ppm were evaluated across volunteers and are presented in Figure 6 (see Supporting Information Tables S3‐S5 for detailed averaged MTR_asym_ contrasts per subject). The intended rotation in scenario Motion‐NoMoco resulted in a strong increase of contrast variability, particularly at Δω = 3 ± 0.25 ppm. The correction of motion (ie, Motion‐Moco) resulted in an underestimation of contrast at 3 ppm and overestimation at 1 and 2 ppm, consistently with the results shown in Figures 4 and 5. The variability of the contrast increased for frequencies farther from 0 ppm. The assumption of variance homogeneity was not met and Games‐Howell’s post‐hoc correction was selected for the multiple‐comparison test with respect to scenario Motion‐MoSensco. The dynamic ${\mathrm{\Delta B}}_{1}^{‐}$ correction step (ie, Motion‐MoSensco vs. Motion‐Moco) resulted in a significant improvement for all frequencies when intended motion was executed (see 5th row in Figure 6). In contrast, the intended head motion (executed mostly while acquiring the Z‐spectral points in the range between 2.75–3.25 ppm, as shown in Figure 4) had a lower impact for offsets acquired at a later time point (left column in Figure 6). Therefore, no significant effect was found for the motion correction at Δω = 1 ± 0.25 ppm (ie, Motion‐MoSensco vs. Motion‐noMoco).

The spatial distribution of motion‐induced artifacts in the CEST contrast among subjects is compared via histograms for ROIs at all 3 different locations (Figure 7). For those regions further from the axes of rotation (ie, ROI‐1 and ROI‐2) the bias in MTR_asym_ became stronger. Motion correction alone decreased the range of observed MTR_asym_ values (ie, Motion‐Moco); whereas additional dynamic ${\mathrm{\Delta B}}_{1}^{‐}$ correction (ie, Motion‐MoSensco) led to a further recovery of mean MTR_asym_ values when compared to the 3 static scenarios. The results of the multiple‐comparison with Games‐Howell correction confirmed that the fully corrected scenario (ie, Motion‐MoSensco) only deviated significantly from Motion‐noMoco and Motion‐Moco with respect to MTR_asym_ values for regions experiencing a larger displacement relative to the coil elements (ie, ROI‐1 and ROI‐2).

## 4 DISCUSSION

In this work, we have investigated the applicability of vNavs for correction of 2 types of motion‐induced artifacts, (1) subject motion and (2) temporal changes in receiver coil sensitivities (${\mathrm{\Delta B}}_{1}^{‐}$). Previous vNav studies tackled the mitigation of subject motion simultaneously with a third type of motion‐induced artifacts (ie, B_0_ fluctuations),^27^, ^28^, ^29^, ^33^, ^54^ whereas we opted for a self‐corrected dynamic ΔB_0_ method directly from the phase of the pre‐saturated GRE images.^43^ Studies combining the correction of these 3 types of artifacts have only recently emerged.^5^, ^8^ Herz et al^8^ managed them retrospectively in the following way:

1. Correction of motion: they applied 3D rigid registration of CEST volumes.^55^, ^56^, ^57^ In contrast, we chose a real‐time approach to track and correct head motion. The main advantage of prospective methods is the direct measurement of the desired k‐space data, avoiding estimation errors because of missing k‐space data.^20^, ^46^ Additionally, the combination of prospective and retrospective methods can be advantageous to correct residual errors of the prospective system.^17^, ^47^
2. Correction of temporal changes in receiver coil sensitivities: a dynamic normalization factor was derived from interleaved M_0_ magnitude acquisitions and further applied to the dynamic glucose‐enhanced (DGEρ) contrast metric. Therefore, the signal variations attributed to relative positional changes between the head and the coil elements were mitigated.^8^ In our study, we chose an analogous retrospective method to compensate the intensity fluctuations of the pre‐saturated GRE images. However, the calculation of this correction factor was based on the data already generated by the interleaved vNavs.^26^ The expected advantage of this approach is the fast acquisition of vNavs, which are able to characterize the spatially smooth $B_{1}^{‐}$ changes with high SNR and apply these on high‐resolution CEST‐labeled images.
3. Correction of temporal B_0_ variations: it was based on the phase data of interleaved unsaturated scans (M_0_).^42^ Instead, our approach retrieved B_0_ maps dynamically from the intrinsically available phase information provided by dual‐echo CEST image readouts. In this way, there was no time delay between the B_0_ estimation and its application to the CEST data.^43^

Compared to the method proposed by Herz et al,^8^ we expect better performance of the presented ΔB_0_ and ${\mathrm{\Delta B}}_{1}^{‐}$ corrections. Our self‐corrected dynamic ΔB_0_ mapping involves field mapping for each Z‐spectral point. In contrast, the method by Herz et al^8^ has a delay with respect to the GRE image acquisitions. The correction of 4 consecutive Z‐spectral points is based on an interleaved M_0_ acquisition. Our r$B_{1}^{‐}$ mapping using vNavs corrects for sensitivity changes with a higher temporal resolution (every Z‐spectral point and not, for example, 4 Z‐spectral points based on the interleaved M_0_ acquisition every ~31 s) and with a constant time delay of ~1.3 s before each readout versus a variable delay depending on the number of Z‐spectral points that are acquired between consecutive M_0_ acquisitions (eg, up to ~31 s delay).

To our knowledge, the current work is the first approach to compensate CEST quantification artifacts, which are related to time‐varying ${\mathrm{\Delta B}}_{1}^{‐}$, via navigators. This is combined with several other established correction steps including ${\mathrm{\Delta B}}_{1}^{+}$ compensation,^41^ dynamic ΔB_0_ compensation,^43^ and real‐time rigid motion correction.^26^ This processing pipeline for CEST quantification provides improved robustness against motion‐induced artifacts. The main finding of this work is that adequate suppression of erroneous signal intensity variations because of non‐uniform receiver coil sensitivities can be achieved from fast vNav scans with lower spatial resolution.

### 4.1 CEST quantification: effect of motion and the proposed correction of motion‐induced artifacts

Real‐time motion correction based on vNavs detects and compensates subject movements that occur during the recovery period between subsequent CEST labeling blocks. However, motion during vNav acquisition, saturation and CEST image sampling cannot be prospectively corrected. Although the majority of the motion can be assumed to take place during the initial, longer delay (9.0 s vs. ~2.5 s), additional retrospective in‐plane rigid registration of the CEST images was proposed to extend the correction to the time frame between the 2nd vNav and CEST acquisition (10.3 s vs. 1.2 s). In this way, motion artifacts arising from accumulated rotations up to 9° were substantially reduced despite contrast differences appreciable in areas close to the borders of the brain, as visible between the static and fully corrected “Dynamic MoSensco” scenarios. There may be residual out‐of‐slice misalignment of consecutive CEST measurements because of motion in the time frame between the 2nd vNav and CEST acquisition (~1.3 s from each other). This can be resolved in the future using 3D image encoding for CEST, such as the Snapshot‐CEST sequence proposed by Zaiss et al,^5^, ^58^ which will allow better retrospective co‐registration.

When prospective motion correction was applied (ie, Static‐Moco, Static‐MoSensco, Motion‐Moco and Motion‐MoSensco), the estimation of motion given by PACE from the 2nd vNav was used to update the position/orientation of the CEST slice. Therefore, the following CEST images, on which the ${\mathrm{\Delta B}}_{1}^{‐}$ correction was performed, may be oriented slightly differently to the preceding 2nd vNav, if some small motion occurred in the ~1.3 s time interval between them. The incorporated smoothing step for the computation of relative r$B_{1}^{‐}$ maps mitigated this effect, and its accuracy with respect to the extent of head rotation is presented in Figure 3E. On average for ROI‐1, the deviation of the smoothed signal was 0.36%/° and 0.39%/° for the 9th and 33th measurement, respectively, implying the effective mitigation of misalignments occurring for single‐step rotations ≤1°. Nevertheless, for larger single step rotation such as the 2.1° rotation corresponding to the 12th measurement (the total accumulated rotation was 8.1°) the remaining deviation increased to 0.99%/°. Therefore, results from CEST measurements on motion‐prone subjects, for which single‐step rotations (between Z‐points acquisitions) are on the order of ~2°, have to be interpreted with care. Immediate reacquisition of corrupted data points as previously proposed by Bogner et al^59^ for MRSI could eliminate this problem. In principle it should be possible to use only a single vNav from which the change in sensitivity can be derived. However, in case of motion this would results in a spatial mismatch of this vNav and the previous vNav, which would require coregistration based on the detected motion before calculation of relative r$B_{1}^{‐}$ maps. This could introduce some errors and is not really necessary, because the dead time for T_1_ recovery before the vNav is anyway unused. For steady‐state CEST (without such a dead time) and 3D‐CEST (requires multiple shots to fill the k‐space) a single vNav option may be useful. The selected voluntary rotation was carried out mostly in the transversal plane to maximize its effects (ie, increased positional variation with respect to the receiver coil elements) and to facilitate its visualization. The effects of different types, degrees, and timing of voluntary motion has not been investigated, representing a potential limitation for its extrapolation. However, we find beforehand no reason to believe that movements of comparable magnitude in other directions would produce results far away from those presented here. The proposed vNav‐CEST MRI acquisition protocol was defined to alternate the offset frequencies from negative to positive sides with respect to water resonance (0 ppm), to minimize the sensitivity of the asymmetric contrast metric to motion. Despite this consideration, the motion‐corrected MTR_asym_ curve shown in Figure 4 (ie, Motion‐Moco case) suffered from strong artifacts not only at Δω = ~3 ppm acquired during the execution of the intended rotations, but also at Δω = ~2 ppm for which the accumulated rotation reached ~7°. The application of the proposed multiple‐correction processing pipeline reduced the MTR_asym_ (3 ± 0.25) deviations relative to the static reference in Figure 4 down to 0.10%, 0.14%, and 0.42% for Δω = 1, 2, and 3 ± 0.25 ppm, respectively.

### 4.2 Statistical analysis

A bias generated by the investigated ${\mathrm{\Delta B}}_{1}^{‐}$ correction could be excluded, regardless of the selected frequency offset or ROI. Nonetheless, a significant effect of the applied motion correction on the CEST contrast was found at Δω = 3 ± 0.25 ppm in absence of voluntary subject movements (ie, Static‐Moco vs. Static noMoco), increasing the ROI‐averaged MTR_asym_ in GM from 5.25% to 5.42%. Although significant, this effect is small and could be related to correction of unavoidable involuntary head motion during the static scans (averaged translation and rotation of 0.46 ± 0.39 mm and 0.17 ± 0.08° across volunteers).

The benefits of correcting temporal ${\mathrm{\Delta B}}_{1}^{‐}$ were clearly demonstrated by Games‐Howell’s post‐hoc tests. Independent of the selected frequency offset (5th row of Figure 6), the fully corrected MTR_asym_ contrast was found to be significantly different from the motion corrected values (ie, Motion‐MoSensco vs. Motion‐Moco) for regions experiencing a larger displacement relative to the coil elements (5th row of Figure 7).

## 5 CONCLUSIONS

We have presented an acquisition protocol that allows the application of a multiple‐correction processing pipeline. Improved robustness of CEST quantification in the presence of motion was demonstrated by comparison of 6 scenarios with and without voluntary head rotations and increasing levels of correction against motion‐induced artifacts. Among them, the fully corrected Motion‐MoSensco confidently achieved restoration of CEST contrast relative to non‐ and/or partially corrected scenarios. It combined 2 correction steps based on 3D EPI volumetric navigators (vNavs): real‐time update of the CEST slice and dynamic mapping of temporal changes in receiver coil sensitivities (${\mathrm{\Delta B}}_{1}^{‐}$). The low‐resolution images generated by the vNav scans allowed the compensation of spatial ${\mathrm{\Delta B}}_{1}^{‐}$, which was identified as a significant source of distortions after the correction of motion. Retrospective ${\mathrm{\Delta B}}_{1}^{‐}$ correction is a viable method, synergistic with the use of vNavs for prospective motion correction that significantly reduce motion‐related artifacts in CEST MRI. In combination with dynamic ΔB_0_ and ${\mathrm{\Delta B}}_{1}^{+}$ corrections, the proposed pipeline may prove to be beneficial in motion prone patients.

## Supporting information


**FIGURE S1** Structural similarity (SSIM) comparison on MTR_asym_ (3 ± 0.25 ppm) weighted maps for volunteer 2. The SSIM maps and global index are calculated between the reference Static‐noMoco (H) and scenarios: Static‐ MoSensco (B); Motion‐noMoco (C); Motion‐Moco (F); and Motion‐MoSensco (G). The rotations tracked are shown in the left and right sides (A, D, E)
**TABLE S1** Global Structural Similarity (SSIM) index comparison among all five volunteers. The measurement is done for the MTR_asym_ (3 ± 0.25 ppm) weighted maps between different scenarios with respect to the uncorrected Static‐noMoco
**TABLE S2** Mean squared error (MSE) index comparison among all five volunteers. The measurement is done for the MTR_asym_ (3±0.25 ppm) weighted maps between different scenarios with respect to the uncorrected Static‐noMoco
**TABLE S3** CEST contrast around 1 ppm expressed as Mean ± Std. error for each volunteer. The calculation considers all voxels belonging to ROI‐1 located in the right frontal lobe of each subject
**TABLE S4** CEST contrast around 2 ppm expressed as Mean ± Std. error for each volunteer. The calculation considers all voxels belonging to ROI‐1 located in the right frontal lobe of each subject
**TABLE S5** CEST contrast around 3 ppm expressed as Mean ± Std. error for each volunteer. The calculation considers all voxels belonging to ROI‐1 located in the right frontal lobe of each subject
