# Supplementary material for: Real‐time motion and retrospective coil sensitivity correction for CEST using volumetric navigators (vNavs) at 7T
Source: Magn Reson Med. 2020 Nov 9;85(4):1909–23. doi: 10.1002/mrm.28555 (PMC7839562; doi:10.1002/mrm.28555)
Supplement: Supplementary file 1 — FIGURE S1 Structural similarity (SSIM) comparison on MTRasym (3 ± 0.25 ppm) weighted maps for volunteer 2. The SSIM maps and global index are calculated between the reference Static‐noMoco (H) and scenarios: Static‐ MoSensco (B); Motion‐noMoco (C); Motion‐Moco (F); and Motion‐MoSensco (G). The rotations tracked are shown in the left and right sides (A, D, E) TABLE S1 Global Structural Similarity (SSIM) index comparison among all five volunteers. The measurement is done for the MTRasym (3 ± 0.25 ppm) weighted maps between different scenarios with respect to the uncorrected Static‐noMoco TABLE S2 Mean squared error (MSE) index comparison among all five volunteers. The measurement is done for the MTRasym (3±0.25 ppm) weighted maps between different scenarios with respect to the uncorrected Static‐noMoco TABLE S3 CEST contrast around 1 ppm expressed as Mean ± Std. error for each volunteer. The calculation considers all voxels belonging to ROI‐1 located in the right frontal lobe of each subject TABLE S4 CEST contrast around 2 ppm expressed as Mean ± Std. error for each volunteer. The calculation considers all voxels belonging to ROI‐1 located in the right frontal lobe of each subject TABLE S5 CEST contrast around 3 ppm expressed as Mean ± Std. error for each volunteer. The calculation considers all voxels belonging to ROI‐1 located in the right frontal lobe of each subject [file MRM-85-1909-s001.docx]

Supporting Information

Real-time motion and retrospective coil sensitivity correction for CEST using volumetric navigators (vNavs) at 7T

## Metrics for Image Quality assessment

In addition to the visual inspection of the motion-induced artifacts noticeable in the MTR_asym_ maps, image quality metrics can be derived for a more quantitative evaluation of the distortions and restoring capability of the proposed correction steps between scenarios outside of the selected ROIs. The following image quality metrics have been calculated using MATLAB (R2017b, MathWorks, Natick, MA, USA):

**Structural Similarity (SSIM)**

This index is a multiplicative combination of local image luminance, structure and contrast. After for luminance and contrast normalization, patterns of pixel intensities are translated into structures among neighboring pixels. Because this metric is computed locally, a quality map can be additionally generated. A value of 0 indicates no structural similarity and 1 in case of two identical data sets.

**Mean squared error (MSE)**

MSE is a risk function that calculates the average squared difference between pixel values from two images. It is always positive and the closer to zero the higher is their similarity.

Supporting Information Tables S1 and S2 show clear effects of the artifacts caused by uncorrected voluntary motion (second row) as well as the improvement provided by our motion correction step across all volunteers for both metrics (i.e, SSIM and MSE) (third row). However the effect of correcting more localized motion-induced B_1_^-^ changes is less obvious as indicated by the low impact on the global SSIM (fourth row).

As seen in Supporting Information Table S1, the SSIM values for the Motion-MoSensco case (fourth row) are not that sensitive to ΔB_1_^-^ correction step (compared to Motion-Moco in the third row). However this global index can be used to discard inferior performance of the proposed processing pipeline across all five subjects and even show a slight overall improvement for two of them after ΔB_1_^-^ correction.

| **SSIM[MTR_asym_ (3±0.25 ppm)]** | **Volunteer** | | | | | **Average** |
| --- | --- | --- | --- | --- | --- | --- |
|  | **1** | **2** | **3** | **4** | **5** |  |
| Static-MoSensco vs Static-noMoco | 0.81 | 0.84 | 0.91 | 0.83 | 0.87 | 0.85 |
| Motion-noMoco vs Static-noMoco | 0.65 | 0.71 | 0.76 | 0.70 | 0.76 | 0.72 |
| Motion-Moco vs Static-noMoco | 0.75 | 0.84 | 0.90 | 0.81 | 0.87 | 0.83 |
| Motion-MoSensco vs Static-noMoco | 0.76 | 0.86 | 0.90 | 0.81 | 0.87 | 0.84 |

Supporting Information Table S1: Global Structural Similarity (SSIM) index comparison among all five volunteers. The measurement is done for the MTR_asym_ (3±0.25 ppm) weighted maps between different scenarios with respect to the uncorrected Static-noMoco.

The MSE metric is presented for each volunteer in Supporting Information Table S2. Despite the higher MSE value for volunteer 1, the averaged MSE was lower for the Motion-MoSensco case than for Motion-Moco (fourth and third row respectively), implying closer MTR_asym_ maps with respect to the reference after ΔB_1_^-^ correction.

| **MSE[MTR_asym_ (3±0.25 ppm)]** | **Volunteer** | | | | | **Average** |
| --- | --- | --- | --- | --- | --- | --- |
|  | **1** | **2** | **3** | **4** | **5** |  |
| Static-MoSensco vs Static-noMoco | 1.29 | 0.42 | 0.10 | 1.48 | 0.16 | 0.69 |
| Motion-noMoco vs Static-noMoco | 52.22 | 14.40 | 1.43 | 10.16 | 2.10 | 16.06 |
| Motion-Moco vs Static-noMoco | 14.69 | 1.31 | 0.10 | 2.58 | 0.14 | 3.77 |
| Motion-MoSensco vs Static-noMoco | 14.86 | 0.86 | 0.08 | 1.80 | 0.12 | 3.54 |

Supporting Information Table S2: Mean squared error (MSE) index comparison among all five volunteers. The measurement is done for the MTR_asym_ (3±0.25 ppm) weighted maps between different scenarios with respect to the uncorrected Static-noMoco.

Supporting Information Figure S1 shows SSIM quality maps relative to the uncorrected static scenario (Static-noMoco) next to the rotation tracked by the vNavs for volunteer 2. Besides the global SSIM index, a visual assessment of scenario Motion-noMoco shows strong structural differences in the frontal region (hypointensities at the top of subfigure C). Even though the motion correction step revealed clear local structural differences in the right frontal lobe (left hand side of subfigure F), the global SSIM index does not present an appreciable difference with the reference scenario Static-noMoco. In the same way, the effect of the proposed dynamic ΔB_1_^-^ correction step (subfigure G) has very small impact on the global index.

Supporting Information Figure S1: Structural similarity (SSIM) comparison on MTRasym (3±0.25 ppm) weighted maps for volunteer 2. The SSIM maps and global index are calculated between the reference Static-noMoco (H) and scenarios: Static- MoSensco (B); Motion-noMoco (C); Motion-Moco (F); and Motion-MoSensco (G). The rotations tracked are shown in the left and right sides (A, D, E).

According to these observations, we can conclude that ROI-based analysis is more appropriate to assess the efficiency of the proposed ΔB_1_^-^ correction step. This is in good agreement with the statistical results presented in Figure 7, where a significant gain was not detectable in regions experiencing mild displacements relative to the coil elements (e.g., ROI-3)

## Summary statistics

To have a closer look at how the presented pipeline performed for each of the subjects, the averaged MTR_asym_ contrasts have been derived for ROI-1 (representative for a region approaching a coil element as rotation takes place). This information is presented in Supporting Information Tables S3, S4 and S5 for frequency ranges Δω= 1,2,3 ± 0.25 ppm respectively.

| **MTR_asym_**  **(1±0.25 ppm)** | **Volunteer** | | | | |
| --- | --- | --- | --- | --- | --- |
|  | **1** | **2** | **3** | **4** | **5** |
| **Static-noMoco** | 4.29 ± 0.07 % | 5.02 ± 0.07 % | 4.26 ± 0.10 % | 4.44 ± 0.09 % | 5.55 ± 0.12 % |
| **Static-Moco** | 4.41 ± 0.09 % | 5.10 ± 0.07 % | 4.31 ± 0.09 % | 4.44 ± 0.12 % | 5.54 ± 0.11 % |
| **Static-MoSensco** | 4.48 ± 0.09 % | 5.12 ± 0.07 % | 4.43 ± 0.09 % | 4.47 ± 0.12 % | 5.67 ± 0.11 % |
| **Motion-noMoco** | 3.67 ± 0.09 % | 5.00 ± 0.16 % | 4.42 ± 0.11 % | 4.10 ± 0.11 % | 5.71 ± 0.19 % |
| **Motion-Moco** | 5.41 ± 0.14 % | 6.24 ± 0.10 % | 4.31 ± 0.06 % | 5.76 ± 0.06 % | 5.72 ± 0.10 % |
| **Motion-MoSensco** | 4.58 ± 0.11 % | 4.71 ± 0.07 % | 4.32 ± 0.06 % | 4.14 ± 0.04 % | 5.17 ± 0.09 % |

Supporting Information Table S3: CEST contrast around 1 ppm expressed as Mean ± Std. error for each volunteer. The calculation considers all voxels belonging to ROI-1 located in the right frontal lobe of each subject.

| **MTR_asym_**  **(2±0.25 ppm)** | **Volunteer** | | | | |
| --- | --- | --- | --- | --- | --- |
|  | **1** | **2** | **3** | **4** | **5** |
| **Static-noMoco** | 6.79 ± 0.08 % | 7.32 ± 0.09 % | 6.91 ± 0.12 % | 6.76 ± 0.15 % | 7.73 ± 0.17 % |
| **Static-Moco** | 6.80 ± 0.08 % | 7.57 ± 0.07 % | 6.84 ± 0.12 % | 6.78 ± 0.10 % | 7.72 ± 0.16 % |
| **Static-MoSensco** | 6.91 ± 0.09 % | 7.53 ± 0.07 % | 6.99 ± 0.12 % | 6.76 ± 0.11 % | 7.62 ± 0.16 % |
| **Motion-noMoco** | 6.43 ± 0.12 % | 7.96 ± 0.16 % | 6.88 ± 0.17 % | 6.79 ± 0.19 % | 8.09 ± 0.24 % |
| **Motion-Moco** | 8.12 ± 0.10 % | 9.75 ± 0.07 % | 6.54 ± 0.07 % | 8.71 ± 0.09 % | 8.07 ± 0.15 % |
| **Motion-MoSensco** | 6.78 ± 0.07 % | 7.27 ± 0.05 % | 6.53 ± 0.07 % | 6.62 ± 0.06 % | 7.54 ± 0.13 % |

| **MTR_asym_**  **(3±0.25 ppm)** | **Volunteer** | | | | |
| --- | --- | --- | --- | --- | --- |
|  | **1** | **2** | **3** | **4** | **5** |
| **Static-noMoco** | 5.39 ± 0.06 % | 5.59 ± 0.05 % | 5.28 ± 0.11 % | 5.04 ± 0.15 % | 4.94 ± 0.13 % |
| **Static-Moco** | 5.50 ± 0.07 % | 5.75 ± 0.06 % | 5.38 ± 0.12 % | 5.39 ± 0.07 % | 5.40 ± 0.14 % |
| **Static-MoSensco** | 5.49 ± 0.06 % | 5.94 ± 0.06 % | 5.47 ± 0.12 % | 5.38 ± 0.07 % | 5.26 ± 0.13 % |
| **Motion-noMoco** | 6.90 ± 0.30 % | 6.07 ± 0.50 % | 5.62 ± 0.57 % | 5.96 ± 0.53 % | 5.44 ± 0.31 % |
| **Motion-Moco** | 2.52 ± 0.16 % | 1.42 ± 0.16 % | 5.37 ± 0.07 % | 1.54 ± 0.14 % | 5.13 ± 0.11 % |
| **Motion-MoSensco** | 5.07 ± 0.10 % | 5.60 ± 0.10 % | 5.22 ± 0.07 % | 4.84 ± 0.13 % | 5.09 ± 0.11 % |

Supporting Information Table S4: CEST contrast around 2 ppm expressed as Mean ± Std. error for each volunteer. The calculation considers all voxels belonging to ROI-1 located in the right frontal lobe of each subject.

Supporting Information Table S5: CEST contrast around 3 ppm expressed as Mean ± Std. error for each volunteer. The calculation considers all voxels belonging to ROI-1 located in the right frontal lobe of each subject.
